# Urotensin-II-Mediated Reactive Oxygen Species Generation via NADPH Oxidase Pathway Contributes to Hepatic Oval Cell Proliferation

**DOI:** 10.1371/journal.pone.0144433

**Published:** 2015-12-11

**Authors:** XiaoTong Yu, PengYan Wang, ZhengMing Shi, Kun Dong, Ping Feng, HongXia Wang, XueJiang Wang

**Affiliations:** 1 Department of Physiology and Pathophysiology, School of Basic Medical Sciences, Capital Medical University, Beijing, China; 2 Beijing Key Laboratory for Cancer Invasion and Metastasis Research, Capital Medical University, Beijing, China; 3 Department of Pathology, Peking Union Medical Hospital, Beijing, China; 4 Department of General Surgery, Beijing Jishuitan Hospital, Beijing, China; 5 Department of Pathology, Beijing Youan Hospital, Capital Medical University, Beijing, China; Chang Gung University, TAIWAN

## Abstract

Urotensin II (UII), a somatostatin-like cyclic peptide, is involved in tumor progression due to its mitogenic effect. Our previous study demonstrated that UII and its receptor UT were up-regulated in human hepatocellular carcinoma (HCC), and exogenous UII promoted proliferation of human hepatoma cell line BEL-7402. Hepatic progenitor cell (HPCs) are considered to be one of the origins of liver cancer cells, but their relationship with UII remains unclear. In this work, we aimed to investigate the effect of UII on ROS generation in HPCs and the mechanisms of UII-induced ROS in promoting cell proliferation. Human HCC samples were used to examine ROS level and expression of NADPH oxidase. Hepatic oval cell line WB-F344 was utilized to investigate the underlying mechanisms. ROS level was detected by dihydroethidium (DHE) or 2’, 7’-dichlorofluorescein diacetate (DCF-DA) fluorescent probe. For HCC samples, ROS level and expression of NADPH oxidase were significantly up-regulated. *In vitro*, UII also increased ROS generation and expression of NADPH oxidase in WB-F344 cells. NADPH oxidase inhibitor apocynin pretreatment partially abolished UII-increased phosphorylation of PI3K/Akt and ERK, expression of cyclin E/cyclin-dependent kinase 2. Cell cycle was then analyzed by flow cytometry and UII-elevated S phase proportion was inhibited by apocynin pretreatment. Finally, bromodeoxyuridine (Brdu) incorporation assay showed that apocynin partially abolished UII induced cell proliferation. In conclusion, this study indicates that UII-increased ROS production via the NADPH oxidase pathway is partially associated with activation of the PI3K/Akt and ERK cascades, accelerates G1/S transition, and contributes to cell proliferation. These results showed that UII plays an important role in growth of HPCs, which provides novel evidence for the involvement of HPCs in the formation and pathogenesis of HCC.

## Introduction

Hepatocellular carcinoma (HCC) is one of among the most commonly occurring cancers worldwide, accounting for >80% of the cases of primary liver tumors [1]. The incidence of HCC continues to rise and liver cancer has become one of the leading causes of cancer-related death [2]. Hepatic oval cells (HOCs) are progenitor cells that activate and migrate from the biliary tree to injured liver in response to chronic hepatitis or toxins, giving rise to hepatocytes and biliary epithelial cells for regeneration and repair [3]. However, growing evidence has revealed that liver tumors may derive from HOCs. Wu et al [4] have found that long-term treatment of HOCs with transforming growth factor-β increased their capacity of self-renewal and occurrence of malignant transformation; *in vivo* xenograft assay demonstrated the tumorigenicity of transformed HOCs in nude mice.

Oxidative damage has a close relationship with tumors. Elevated levels of reactive oxygen species (ROS) are found in several human cancers, such as HCC, breast cancer, and prostate cancer [5–7]. NADPH-oxidase-induced ROS generation has been demonstrated to promote cancer cell growth, invasion and angiogenesis [8]. However, the role of NADPH-oxidase-dependent ROS in regulating the biological behavior of HOCs remains unknown.

Urotensin II (UII) is a somatostatin-like cyclic peptide which was initially isolated from the urophysis of teleost fish in the 1960s [9]. Human UII is an undecapeptide (H-Glu-Thr-Pro-Asp-c[Cys-Phe-Trp-Lys-Tyr-Cys]-Val-OH), and orphan G-protein coupled receptor 14, UT, was identified as the urotensin II receptor [10]. Many studies have revealed that mRNA and/or protein of UII/UT are expressed in several human tissues. In human liver, there is a low level of UII mRNA expression [11]. Previously, UII was known as a potent vasoconstrictor that played important roles in cardiovascular disease [12]. Continuing research has shown that UII exerts mitogenic effects on many cell lines, such as pulmonary artery smooth muscle cells (PASMCs) and cardiac fibroblasts. It is reported that UII stimulates membrane-bound NADPH oxidase to generate ROS in promoting PASMC proliferation [13]. To the best of our knowledge, there is no evidence of the relationship between UII and ROS in HOCs.

Recent studies have suggested that the UII/UT system is involved in the pathogenesis of different human tumors. Our previous study demonstrated that UII and UT were up-regulated in rat hepatoprecancerous lesions and human HCC tissue, and exogenous UII increased human hepatoma cell line BEL-7402 proliferation *in vitro* [14,15]. HOCs may be affected by liver microenvironment and high levels of UII possibly mediate their biological characteristics. However, as a carcinogenic factor, the effects of UII on the proliferation of HOCs and its molecular mechanisms need to be clarified.

In this study, we aimed to investigate the effect of UII on the generation of ROS via NADPH oxidase pathways in HOCs, and the role of ROS in promoting cell proliferation and its possible mechanisms.

## Materials and Methods

### Pathological specimens from patients

Ethical approval was obtained from the Ethics Committee of Capital Medical University. All participants provided their verbal informed consent to participate in this study and informed consent was recorded by pathologic doctor. The consent was verbal because the resected human liver tissues were obtained during surgical procedure and no additional treatment for patients. The ethics committees approved this consent procedure.

27 paired human HCC samples were obtained from patients at Beijing YouAn Hospital, Capital Medical University. The adjacent noncancerous tissues were more than 5 cm away from the edge of tumor and both tissues were confirmed by histological diagnosis. According to tumor node metastasis (TNM) classification, patients diagnosed with T2N1M0, without type 2 diabetes mellitus, cardiovascular diseases and hypertension, were selected for this study. The samples tested were consistent with previous samples with UII up-regulation [15].

## Materials

Human UII powder and DHE fluorescent probe were purchased from Sigma–Aldrich (St Louis, MO, USA). The western blotting kit, anti-GADPH antibodies, anti-p40phox antibodies, anti-p47phox antibodies, anti-p67phox antibodies and anti-NOX2 antibodies were purchased from Santa Cruz Biotechnology (Santa Cruz, CA, USA). Antibodies directed against cyclin E, CDK2, PI3K, phospho-PI3K, Akt, phospho-Akt, ERK1/2 and phospho-ERK1/2 were purchased from Cell Signaling Technology (Danvers, MA). The Reactive Oxygen Species Assay Kit was purchased from Nanjing Jiancheng Bioengineering Institute (Nanjing, Jiangsu, China). The bromodeoxyuridine (Brdu) Labeling and Detection Kit was purchased from Roche Diagnostics (Mannheim, Germany). All chemical reagents were of analytical grade.

### Cell culture and treatment

The rat hepatic oval cell line WB-F344 cells were isolated from the liver of an adult male Fischer 344 rat [16]. In this study, WB-F344 cells were obtained from the Cell Bank of the Chinese Academy of Science. Cells was cultured at 37°C and 5% CO_2_ in DMEM supplemented with 10% fetal bovine serum and antibiotics (100 U/mL penicillin and 100μg/mL streptomycin). Medium was replaced with serum-free DMEM for 12 h, and cells were pretreated with or without apocynin (0.5 mM) or urantide (1 mM) for 30 min, and stimulated with UII. Untreated cells were considered as controls.

### Western blot analysis

Protein samples were prepared from human liver tissues and WB-F344 cells, and western blot analysis was performed as described [17]. After centrifugued at 12,000 rpm at 4°C for 30min, 70–80 μg protein lysate was subjected to 10% SDS-PAGE electrophoresis, followed by transfer to the polyvinylidenedifluoride membranes. The membranes were blocked with 5% BSA in TBST for 1 h at room temperature. Primary antibodies against p47phox (1:800), p40phox (1:800), p67phox (1:800), NOX2 (1:800), p-ERK1/2 (1:1000), ERK1/2 (1:1000), p-PI3K (1:1000), PI3K (1:1000), p-Akt (1:1000), Akt (1:1000), cyclin E (1:800) and CDK2 (1:800) were incubated at 4°C overnight, as well as GADPH (1:5000). Membranes were then washed three times with TBST for 15 min and incubated with horseradish-peroxidase-conjugated secondary antibodies (1:5000 dilution). The immunoreactive bands were developed by use of enhanced chemiluminescence (ECL) detection reagents, and scanned with a FlourChem HD2 System (Protein Simple, USA).

### ROS determination

For liver tissues, DHE fluorescent probe was used for detecting ROS generation. Frozen sections were fixed with iced acetone at 4°C for 1 h. The sections were washed with PBS and incubated with 10 μM DHE fluorescent probe for 1 h at 37°C. Tissue images were visualized by a fluorescent microscope (Olympus, Tokyo, Japan). For cells, after UII stimulation with or without apocynin (0.5 mM) or urantide (1 mM) pre-treatment, DCF-DA (10 μM) was added to medium for 30 min, cells were incubated at 37°C in the dark, and then washed immediately with PBS. Cells were trypsinized and washed; 5,000 cells were determined for each sample. The fluorescence emitted at 488 nm was measured by a FACS Calibur flow cytometer (BD Biosciences, San Jose, CA).

### Determination of SOD, GSH-Px and malondialdehyde (MDA) activities

The homogenates of HCC tissue and adjacent noncancerous tissue were obtained from frozen tissue. Homogenates were first centrifuged at 3,000 g for 30 min at 4°C, and then measured the protein concentrations of the supernatants by using a BCA Protein Assay Kit (BioTeke Corporation, China). The level of MDA and the activities of SOD and GSH-Px were determined using colorimetric assays with commercial kits which were performed as described [18].

### Cell cycle assay

Cell cycle analysis was examined by flow cytometry. Cells (10^4^) were harvested and fixed with 75% alcohol at 4°C overnight. After washed with PBS, cells were resuspended in 500 μl RNase (0.02 mg/ml) and incubated at 37°C for 30 min. Then 100μl propidium iodide (0.5 mg/ml) was added and the DNA content of stained nuclei was analyzed by a FACS Calibur flow cytometer (BD Biosciences, San Jose, CA).

### MTT assay

MTT assay was performed as previously described [14]. Briefly, WB-F344 cells were seeded in 96-well plates at 1×10^4^ cells/ml and cultured for 24 h. After 24h starvation, different 10^−10^–10^−6^ M UII was added and cells were cultivated further for 24 h. Then each well was incubated with 20 μl MTT (2.5mg/ml) solution for a further 4 h. After that, cells were lysed with 150 μl dimethyl sulfoxide (DMSO). The absorbance of the final product was measured at 490 nm.

### Cell proliferation assay

Cell proliferation was measured by incorporation of Brdu, as previously described [15]. Cells were seeded in 24-well plates at 1×10^5^/cells. When cells reached 60% confluence, the medium was replaced with serum-free DMEM for 24 h and then stimulated with UII for 24 h. Brdu was added to the medium for the last 1 h. After washed with PBS, cells were fixed with 4% paraformaldehyde for 30 min. Brdu incorporation was determined with a Brdu Labeling and Detection Kit (Roche Diagnostics). Cells were visualized with a fluorescence microscope (20×; Olympus). Brdu-labeled cells and cell nuclei were counted by Image J software (National Institutes of Health, USA). The proportion of Brdu was calculated by number of Brdu-labeled cells/number of cell nuclei × 100%.

### Statistical analysis

Results are expressed as mean ± SEM. Statistical analysis was performed using a two-tailed unpaired Student’s *t* test and one-way analysis of variance followed by a Student–Newman–Keuls test. The SPSS version 12.0 software (SPSS Inc., Chicago, IL, USA) was used for statistical analyses. *P*<0.05 was denoted to be statistically significant.

## Results

### Human HCC tissues showed increased ROS and expression of NADPH oxidase

**Fig 1:**
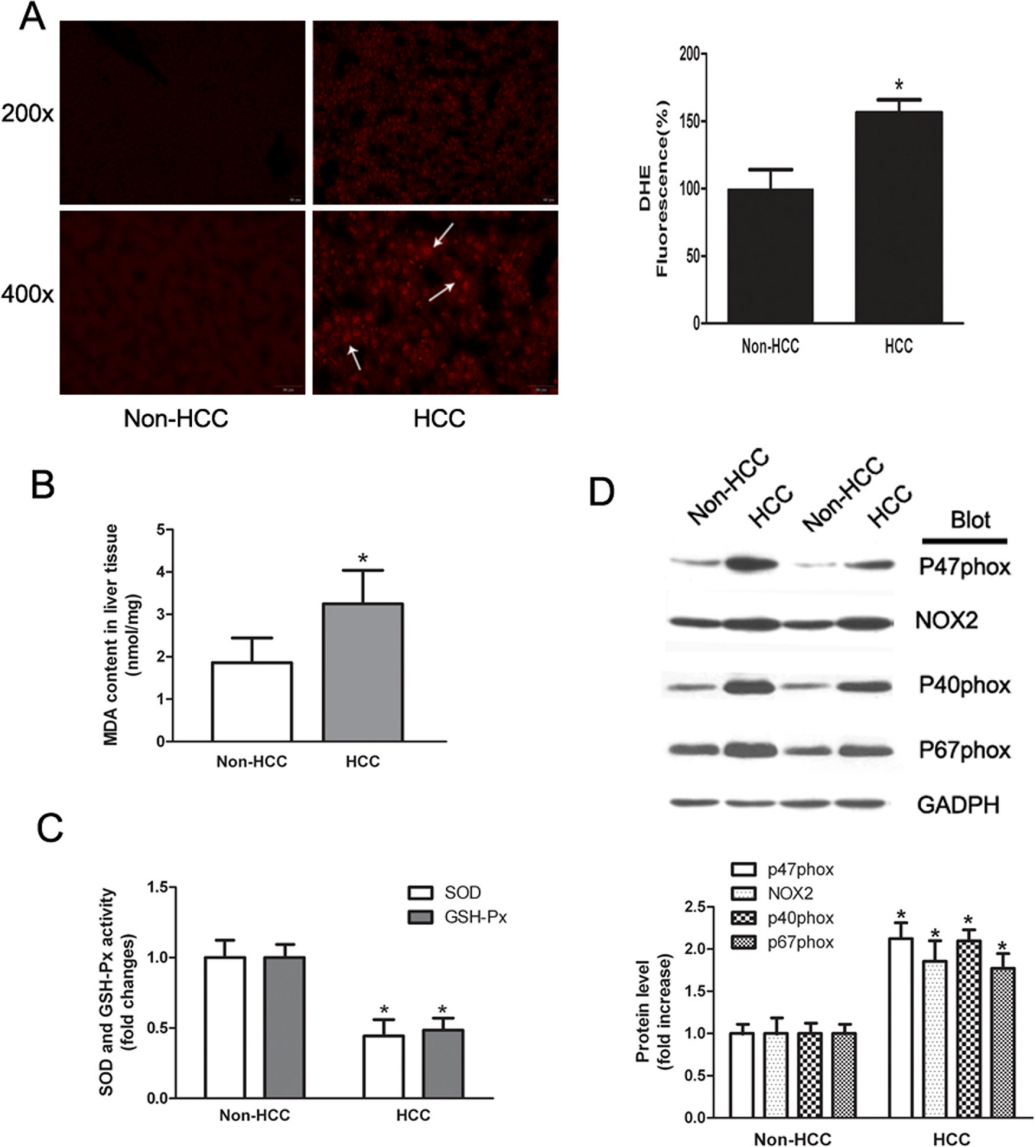
ROS level and expression of NADPH oxidase were elevated in HCC. (A) Fresh human HCC tissues and adjacent noncancerous tissues were stained with DHE. The fluorescence signals were captured by fluorescent microscopy and analyzed by Image J software. (B) Malondialdehyde content in human liver tissue was measured by thiobarbituric acid assay. (C) Activity of superoxide dismutase and glutathione peroxidase was measured by glutathione assay. (D) Expression of NADPH oxidase subunit p47phox, NOX2, p40phox and p67phox was measured by western blotting and normalized against GADPH. The results are presented as mean ± SEM. * indicates significant difference compared to adjacent liver tissues, *P*<0.05.

We used 27 paired human HCC tissues and adjacent noncancerous liver tissues to determine ROS levels by intracellular dye DHE staining. The red area showed ROS signals (white arrow). In HCC regions, the intensity of fluorescence was 1.5–1.7-fold higher compared with the corresponding noncancerous tissues (Fig 1A). The malondialdehyde content in HCC was higher than in noncancerous tissue, and the activity of superoxide dismutase and glutathione peroxidase was decreased (Fig 1B and 1C). The NADPH oxidase complex includes NOX2, p67phox, p47phox, p40phox and p22phox (NOX4), and in endothelial cells, it is considered as an important source of ROS [19]. Here, the expression of NADPH oxidase was examined by western blotting. The protein levels of NOX2, p67phox, p47phox and p40phox were significantly increased in HCC tissues (Fig 1D).

### NADPH oxidase was activated in HOCs depending on UII stimulation

**Fig 2:**
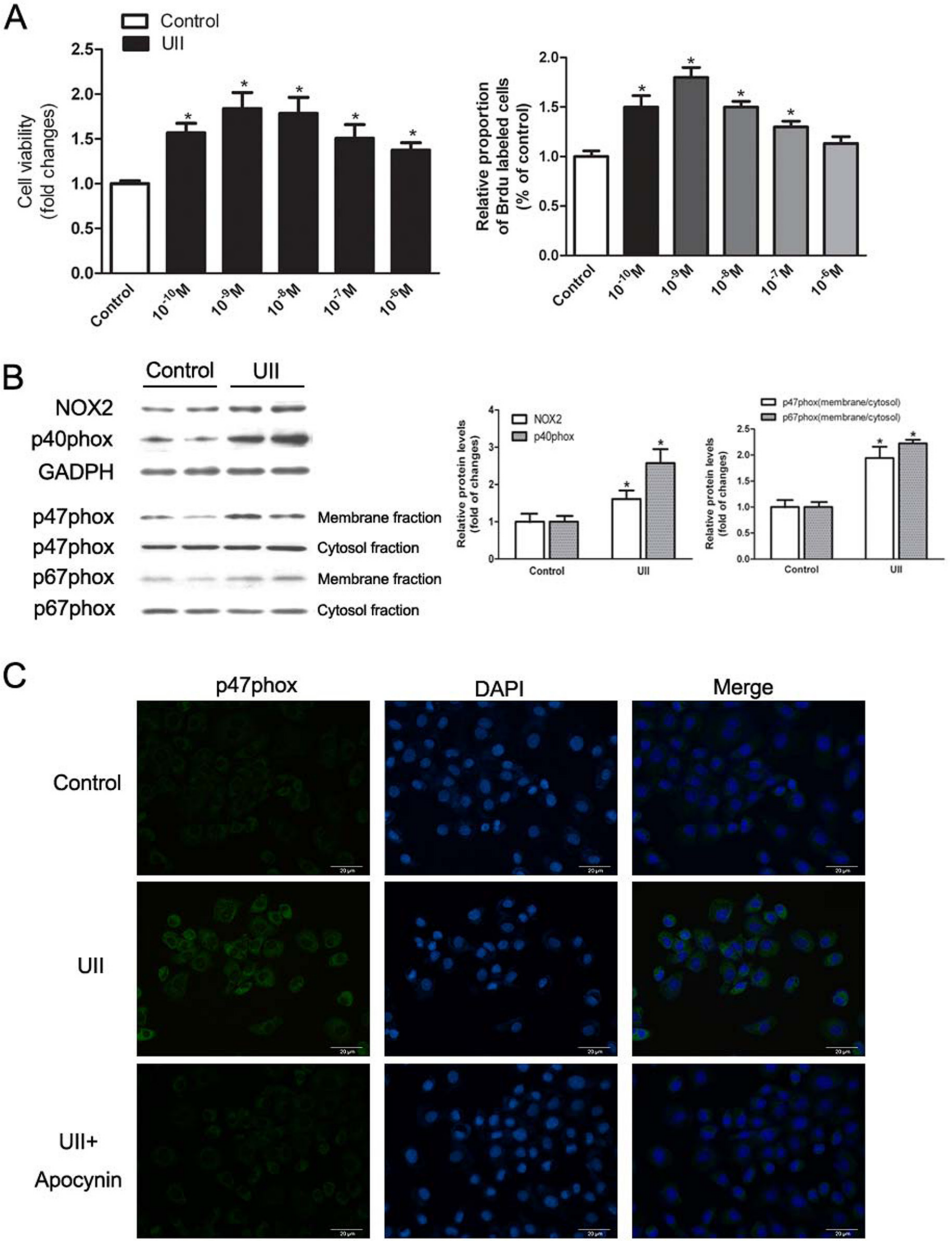
UII increased expression of NADPH oxidase subunits. (A) The optimum concentration of UII was determined by MTT assay and Brdu assay. After 24 h starvation, cells were treated with different 10^−10^–10^−6^ M UII for 24 h. Cell viability was tested by MTT assay. Cell proliferation was detected by Brdu incorporation. The results are presented as mean ± SEM (n = 6). * *P*<0.05 versus control. (B) Expression of NADPH oxidase subunits were measured by western blotting and normalized against GADPH or differences between membrane fraction and cytosol fraction. After starvation, cells were treated with UII (10^−9^ M) for 2 h. Data are presented as mean ± SEM (n = 3). * *P*<0.05 versus control. (C) Immunofluorescence staining detected intracellular protein levels of p47phox upon UII stimulation. After starvation, cells were treated with UII (10^−9^ M) for 2 h with or without apocynin (0.5 mM) pretreated for 30 min. The fluorescence signals were captured by fluorescent microscopy.

Our previous study demonstrated that the UII/UT system was up-regulated in HCC. To investigate whether UII acted on activation of NADPH oxidase and induced ROS production in HOCs, and the role of HOCs in liver tumor formation, we used the WB-F344 cell line. Cells were treated with 10^−10^–10^−6^ M UII for 24 h, then cell viability was measured by MTT assay and Brdu incorporation was utilized to determine cell proliferation. The results showed that UII significantly increased cell viability and proliferation, and 10^−9^ M UII mediated the most mitogenic effect (Fig 2A). The protein levels of NOX2 and p40phox were up-regulated upon UII stimulation. Besides, comparing the membrane fractions to cytosol fractions of p47phox and p67phox, the translocations of proteins were increased in HOCs (Fig 2B). Apocynin, a NADPH oxidase inhibitor, which affects p47phox translocated from cell cytoplasm to membrane. We found 0.5 mM apocynin could inhibit NADPH oxidase dependent ROS generation in oval cells but not affect cell growth (data not shown), so 0.5 mM apocynin was utilized to support our conclusions in this study. Immunofluorescence analysis showed that apocynin pretreatment abolished UII-mediated expression of p47phox (Fig 2C).

### UII elevated ROS generation in WB-F344 cells

**Fig 3:**
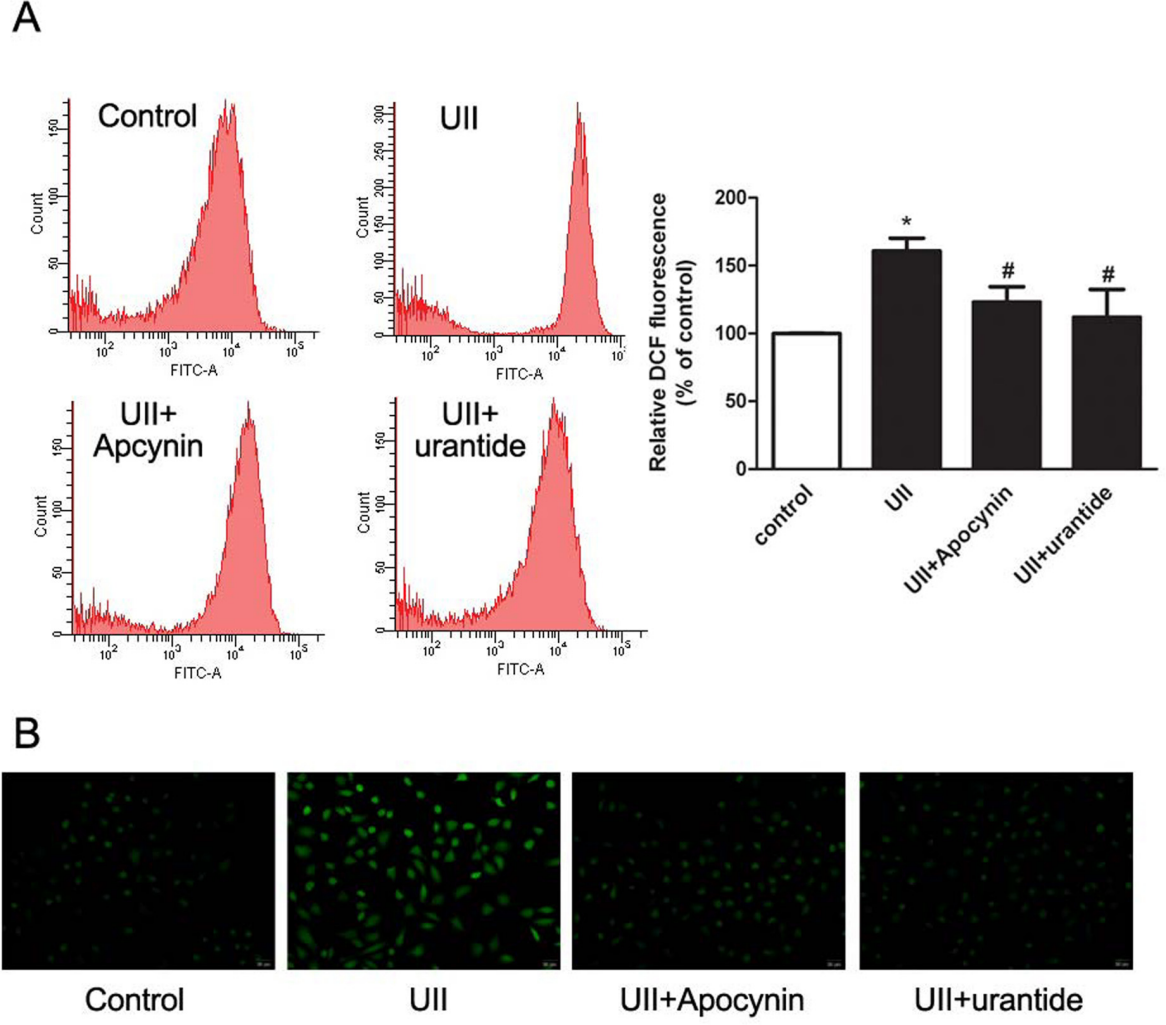
Apocynin or urantide inhibited UII-elevated ROS production. (A) Flow cytometric histogram of DCF in WB-F344 cells. After starvation, cells were treated with UII (10^−9^ M) for 2 h, with or without apocynin (0.5 mM) or urantide (1 mM) pre-treatment for 30 min. The relative DCF fluorescence intensity was determined by flow cytometry. Data are presented as mean ± SEM (n = 6). * *P*<0.05 versus control. # *P*<0.05 versus UII group. (B) ROS signals were captured by fluorescent microscopy.

To investigate whether UII increased ROS level via NADPH oxidase pathways in HOCs, we used DCF fluorescent probe to detect intracellular ROS production. In the UII-treated group, the mean fluorescence intensity, which reflected ROS production, was increased compared with that in untreated cells. Pretreatment with NADPH oxidase inhibitor apocynin or UT antagonist urantide significantly decreased UII-induced ROS level (Fig 3A). In addition, we observed ROS signals using fluorescent microscopy. The green fluorescence represented ROS positivity. The results showed that UII elevated ROS generation and apocynin or urantide partially decreased UII-up-regulated ROS level (Fig 3B).

### Apocynin partially abolished UII-induced phosphorylation of PI3K/Akt and ERK in HOCs

**Fig 4:**
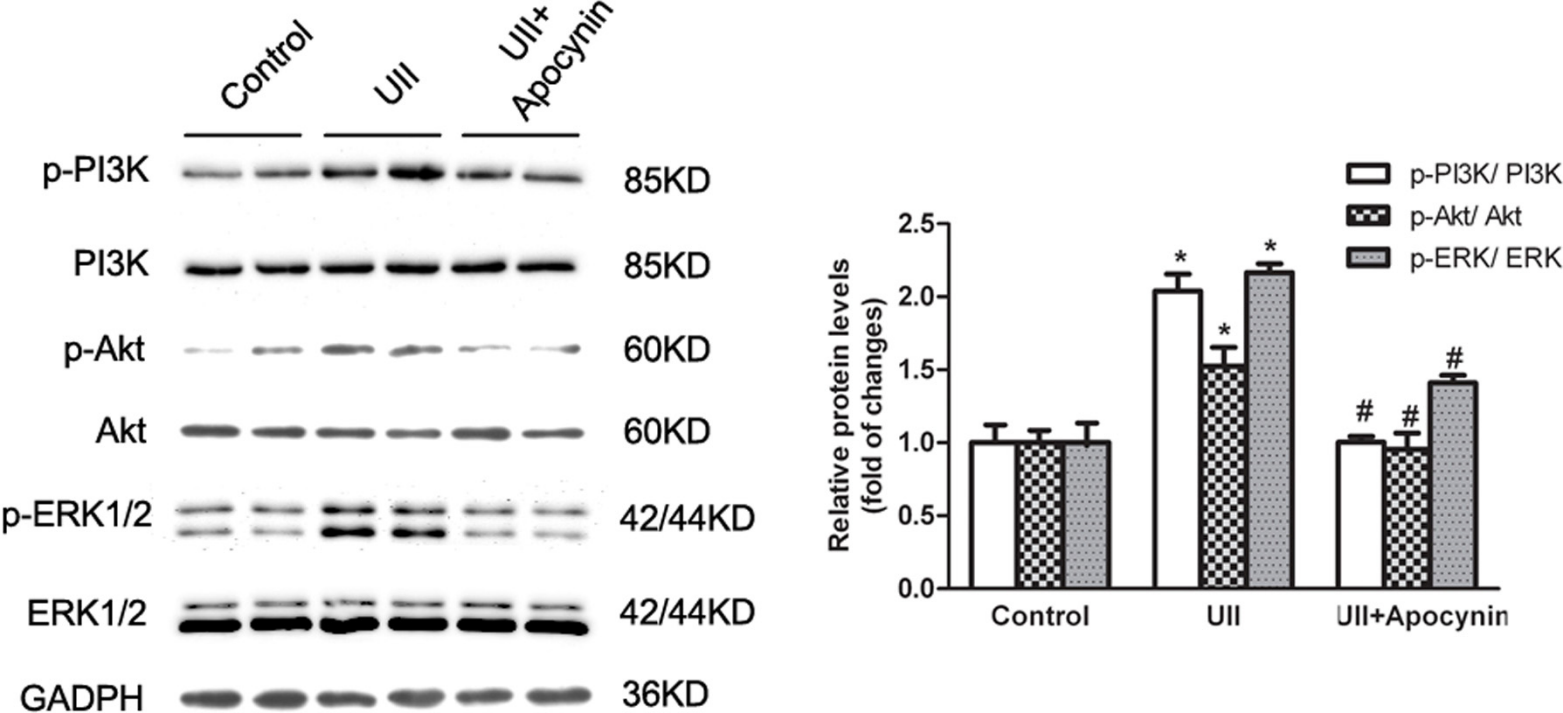
UII-increased phosphorylation of PI3K/Akt and ERK was partially abolished by apocynin. The protein levels were measured by western blotting and normalized against GADPH. After starvation, cells were treated with UII (10^−9^ M) for 2 h. Data are presented as mean ± SEM (n = 6). * *P*<0.05 versus control. # *P*<0.05 versus UII group.

Our previous work showed that UII promoted HOC proliferation through the ERK signaling pathway [14]. It was also suggested that PI3K/Akt was involved in cell proliferation and affected by intracellular ROS level. In order to demonstrate PI3K/Akt and ERK pathway were involved in UII induced WB-F344 cell proliferation, firstly we used LY294002 (a PI3K inhibitor) and PD184352 (an ERK inhibitor) to detect proliferate cells. As shown in S1 Fig, LY294002 and PD184352 pretreatment could inhibit UII induced cell proliferation. Besides, when pretreated with UII receptor antagonist urantide, the phosphorylation of PI3K/Akt and ERK was decreased compared to UII treated cells (S2 Fig). These results revealed that UII could recognize its receptor and then activated PI3K/Akt and ERK pathway to promote cell proliferation. Here, pre-treatment with apocynin decreased UII-induced phosphorylation of PI3K/Akt and ERK in WB-F344 cells. So, UII-elevated ROS generation partially contributed to activation of the PI3K/Akt and ERK cascades, indicating that ROS may play a role in UII-mediated cell proliferation (Fig 4).

### UII-induced ROS generation accelerated G1/S transition and increased cyclin E/CDK2 expression in HOCs

**Fig 5:**
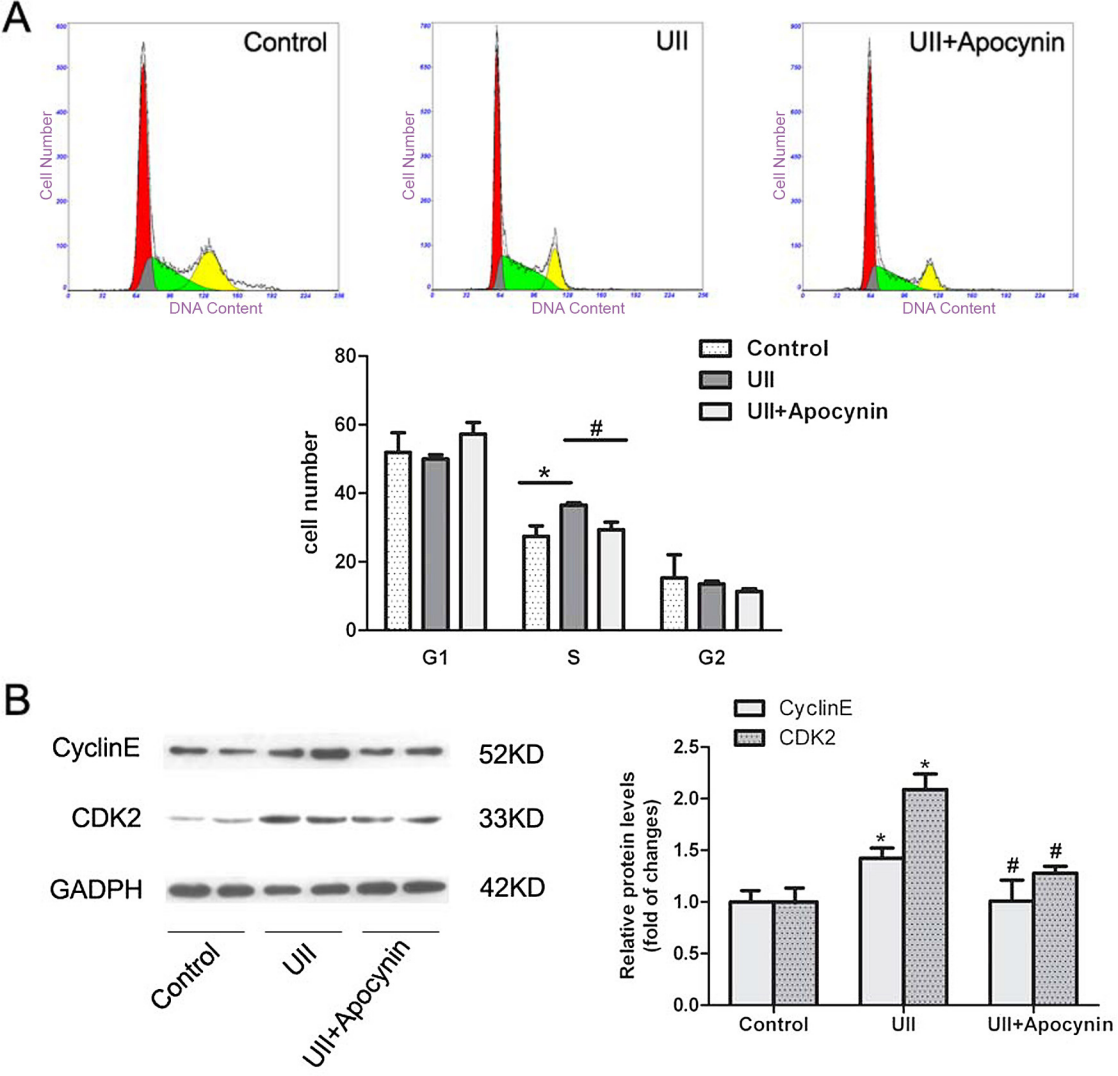
ROS accelerated G1/S transition and increased cyclin E/CDK2 expression in WB-F344 cells. (A) Cell cycle was assessed by flow cytometry. After starvation, cells were treated with UII (10^−9^ M) for 12 h, with or without apocynin (0.5 mM) pre-treatment for 30 min. The results are presented as mean ± SEM (n = 5). * *P*<0.05 versus control. # *P*<0.05 versus UII group. (B) Expression of cyclin E and CDK2 was examined by western blotting and normalized against GADPH. Data are presented as mean ± SEM (n = 3). * *P*<0.05 versus control. # *P*<0.05 versus UII group.

To clarify whether UII-elevated ROS production affected the cell cycle, we determined its effects on the cell cycle distribution by flow cytometry. As shown in Fig 5A, the percentage of UII-treated WB-F344 cells in S phase was increased compared with the control cells. Pre-treatment with apocynin significantly reduced the increase in UII elevation of cells in S phase. To determine whether UII-induced ROS generation affected the level of G1/S transition-regulatory factors, we examined expression of cyclins and CDKs by western blotting. After treatment with UII, the protein levels of cyclin E and CDK2 were up-regulated, while pre-treatment with apocynin decreased expression of cyclin E and CDK2 compared with UII-treated cells (Fig 5B). These results indicated that UII could increased G1/S transition-regulatory proteins and promoted G1/S transition.

### ROS contributed to UII-induced WB-F344 cell proliferation

**Fig 6:**
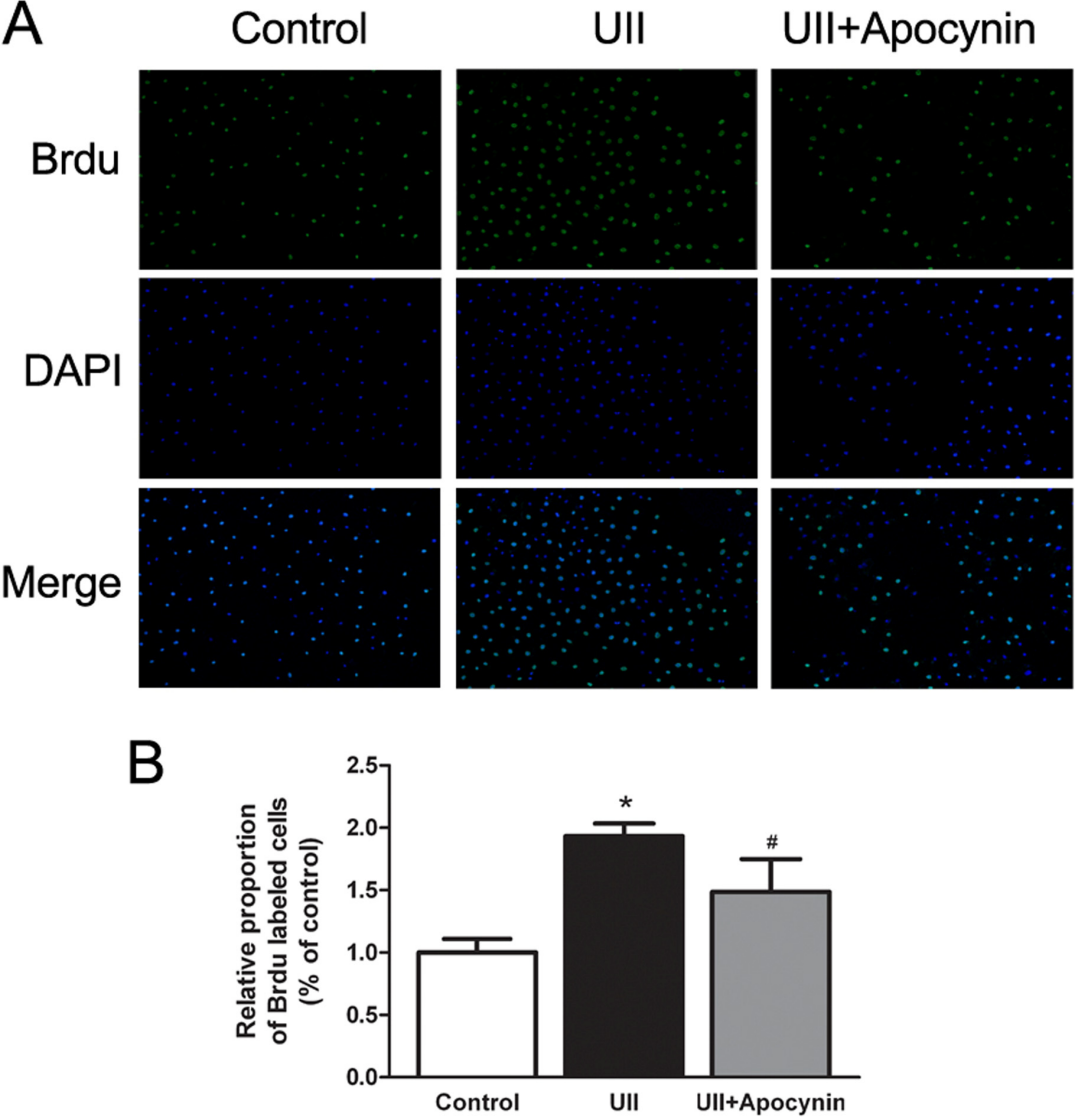
UII-induced ROS production contributes to cell proliferation. (A) Cell proliferation was determined by Brdu incorporation assay. After starvation, cells were treated with UII (10^−9^ M) for 24 h, with or without apocynin (0.5 mM) pre-treatment for 30 min. Data are presented as mean ± SEM (n = 6). *P<0.05 versus control; # *P* < 0.05 versus UII group. (B) The proportion of Brdu-labeled cells was measured by Image J software.

To examine whether production of ROS was required for UII-induced cell proliferation, WB-F344 cells were pretreated with apocynin and Brdu incorporation was analyzed by fluorescence microscopy. As shown in Fig 6, UII-induced WB-F344 cell proliferation was partially abolished by apocynin pre-treatment.

## Discussion

Our present work demonstrated that UII could induce ROS generation via NADPH oxidase pathways in HOCs, which contributed to activation of the PI3K/Akt and ERK signaling pathways and G1/S phase transition, and eventually, cell proliferation. The role of UII in mediating the occurrence of oxidative damage in HOCs may lead to cell malignant transformation, and our results provide new evidence of the role of up-regulation of UII in the formation and development of HCC.

HCC is the fifth most prevalent cancer worldwide, with a 5% survival rate >5 years and > 500,000 deaths annually [20]. Generally, liver cirrhosis is considered to be a precancerous lesion, and factors leading to liver damage promote the development of HCC, such as alcohol abuse, hepatitis virus infection, or metabolic disorders. UII, a vasoactive peptide, is known to act as an autocrine/paracrine growth stimulator, which is highly expressed in many tumors and promotes tumor cell proliferation *in vitro* [21,22]. Our previous study demonstrated that the UII/UT system was up-regulated in HCC tissue. In the present work, we found that ROS level and NADPH oxidase subunits NOX2, p67phox, p47phox and p40phox were increased in UII up-regulated HCC samples (Fig 1). These results suggest that UII induces ROS generation via NADPH oxidase pathways.

As for the origin of liver tumor cells, increasing evidence has revealed that malignant transformation of hepatic progenitor cells (HPCs) is responsible for liver cancer initiation, which is known as the “cancer stem cell hypothesis” [23]. Yi Tang et al found that cells in human hepatocellular cancer labeled hepatic stem cell markers [24]. Besides, hepatitis, alcohol or toxins could target HPCs and leading to cell expansion and even transformation [25]. These findings suggested that HCC may be derived from HPCs and HPCs appeared to be critical to the development of human liver tumor. However, the mechanism of HPC-induced HCC remains unclear. HOCs are considered to be HPCs in rat liver and the WB-F344 cell line is commonly used in various studies. The biological behavior of HOCs is affected by liver microenvironment, and changes in IL-6, tumor necrosis factor-α or other growth factors may lead to excessive proliferation and abnormal differentiation of HOCs [26]. To establish whether high concentration of UII in the liver microenvironment induces HOC proliferation, we stimulated WB-F344 cells with UII and investigated cell proliferation *in vitro*. As shown in Fig 2A, UII significantly increased cell viability and cell proliferation. Meanwhile, we found that UII elevated NADPH oxidase subunit expression and ROS levels in HOCs, and apocynin or urantide partially abolished this effect (Figs 2 and 3). These results revealed that UII induced ROS generation via NADPH oxidase pathways in HOCs.

UII is recognized by its receptor UT and subsequently activates downstream signaling pathways, such as phospholipase C/inositol 1,4,5-triphosphate (PLC/IP3), PKC/ERK and RhoA/Rho kinase [27]. Some redox-sensitive signaling cascades are activated by ROS generation, including PKC/ERK and Akt [28,29]. ROS, NADPH oxidase and redox-sensitive activation of MAPKs are implicated to play an important role in cell proliferation [30,31]. Our previous study demonstrated that UII increased WB-F344 cell proliferation via activation of the PKC and ERK signaling pathways [14]. In the supporting information, we demonstrated that UII recognized its receptor and activated PI3K/Akt and ERK pathway in promoting HOCs proliferation. In the present study, we used NADPH oxidase inhibitor apocynin to examine the effects of UII-induced ROS in activation of PI3K/Akt and ERK, cell cycle distribution, and cell proliferation. Our results suggested that UII-induced ROS generation contributed to the phosphorylation of PI3K/Akt and ERK (Fig 4). In addition, the production of ROS accelerated G1/S phase transition via increased expression of G1/S transition-regulatory factors cyclin E and CDK2 (Fig 5). Finally, Brdu incorporation assay showed that apocynin, which could inhibit NADPH oxidase-dependent ROS generation, decreased UII-induced cell proliferation (Fig 6).

In conclusion, our results support that UII-mediated generation of ROS via NADPH oxidase pathways plays an important role in promoting cell proliferation by activated PI3K/Akt and ERK signaling pathways, and accelerates G1/S phase transition in WB-F344 cells. However, the effect of UII and ROS in HOC malignant transformation needs to be clarified.

## Conclusions

Our study provides further evidence for the important role of UII in HCC, which originates from HPCs. The elevated level of UII in HCC may contribute to oxidative damage in HOCs via the NADPH oxidase pathway and increase HOC proliferation. UII-induced ROS production is partially associated with activation of the PI3K/Akt and ERK signaling pathways and accelerated G1/S transition, which may be the underlying mechanisms of UII-mediated ROS in promoting cell proliferation. UII induces the occurrence of oxidative damage in HOCs and promotes cell proliferation, which may provide the possibility of cell malignant transformation. These findings confirm the important role of UII in progression of HCC, and shed light on new aspects for cancer prevention.

## Supporting Information

S1 Fig UII induced WB-F344 proliferation was inhibited by PD183252 and LY294002. Cell proliferation was determined by Brdu incorporation assay. After starvation, cells were treated with UII (10^−9^ M) for 24 h, with or without PD184352 or LY294002 pre-treatment for 30 min.
(TIF)

S2 Fig Urantide abolished UII-elevated phosphorylation of PI3K/Akt and ERK. The protein levels were measured by western blotting and normalized against GADPH. After starvation, cells were treated with UII (10^−9^ M) for 2 h, with or without urantide pre-treatment for 30 min. Data are presented as mean ± SEM (n = 6). * *P*<0.05 versus control. # *P*<0.05 versus UII group.
(TIF)
