# Supplementary figures and images for: Urotensin-II-Mediated Reactive Oxygen Species Generation via NADPH Oxidase Pathway Contributes to Hepatic Oval Cell Proliferation
Source: PLoS One. 2015 Dec 11;10(12):e0144433. doi: 10.1371/journal.pone.0144433 (PMC4676694; doi:10.1371/journal.pone.0144433)

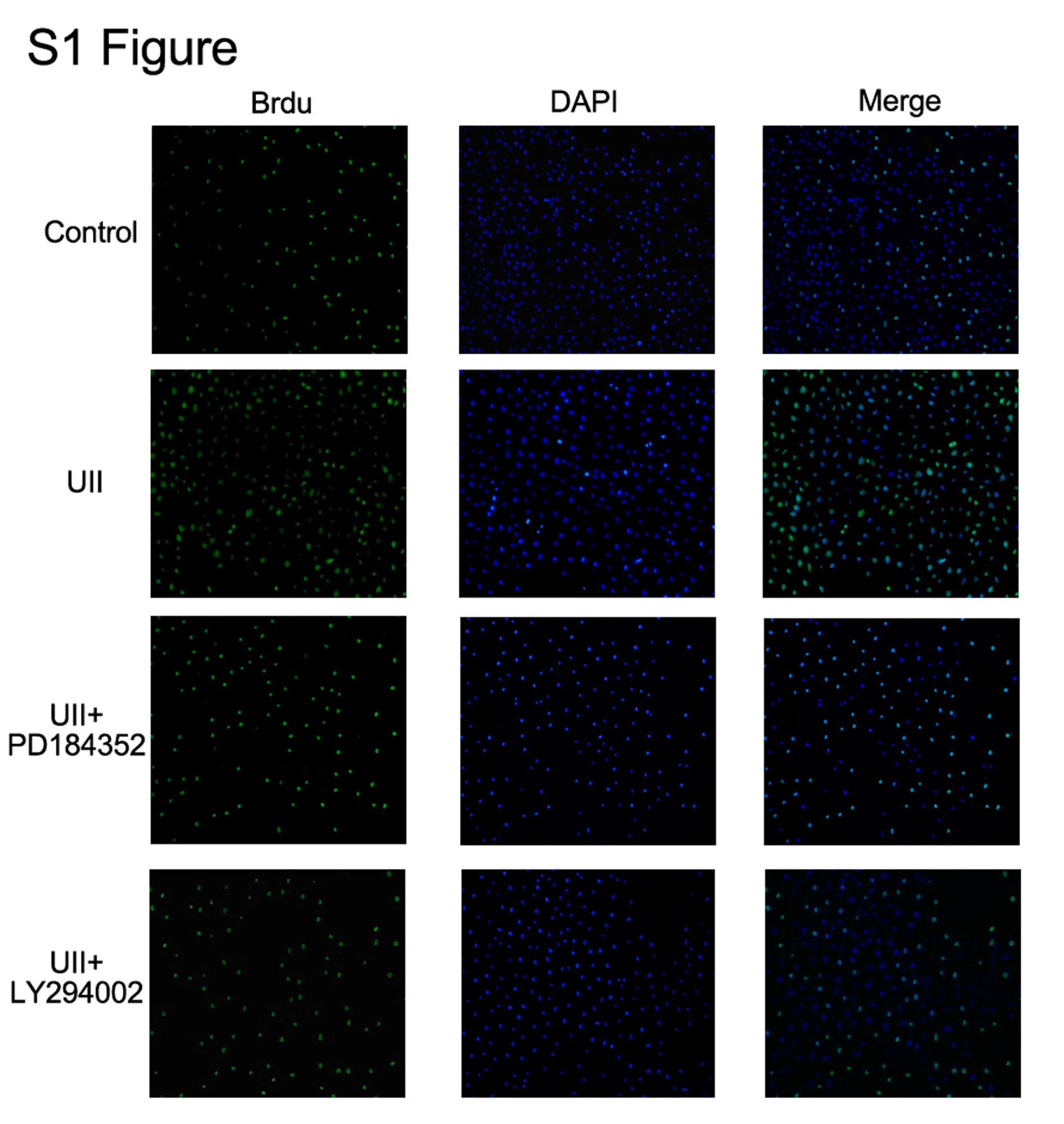

Supplement: S1 Fig — Cell proliferation was determined by Brdu incorporation assay. After starvation, cells were treated with UII (10−9 M) for 24 h, with or without PD184352 or LY294002 pre-treatment for 30 min. (TIF) [file pone.0144433.s001.tif]

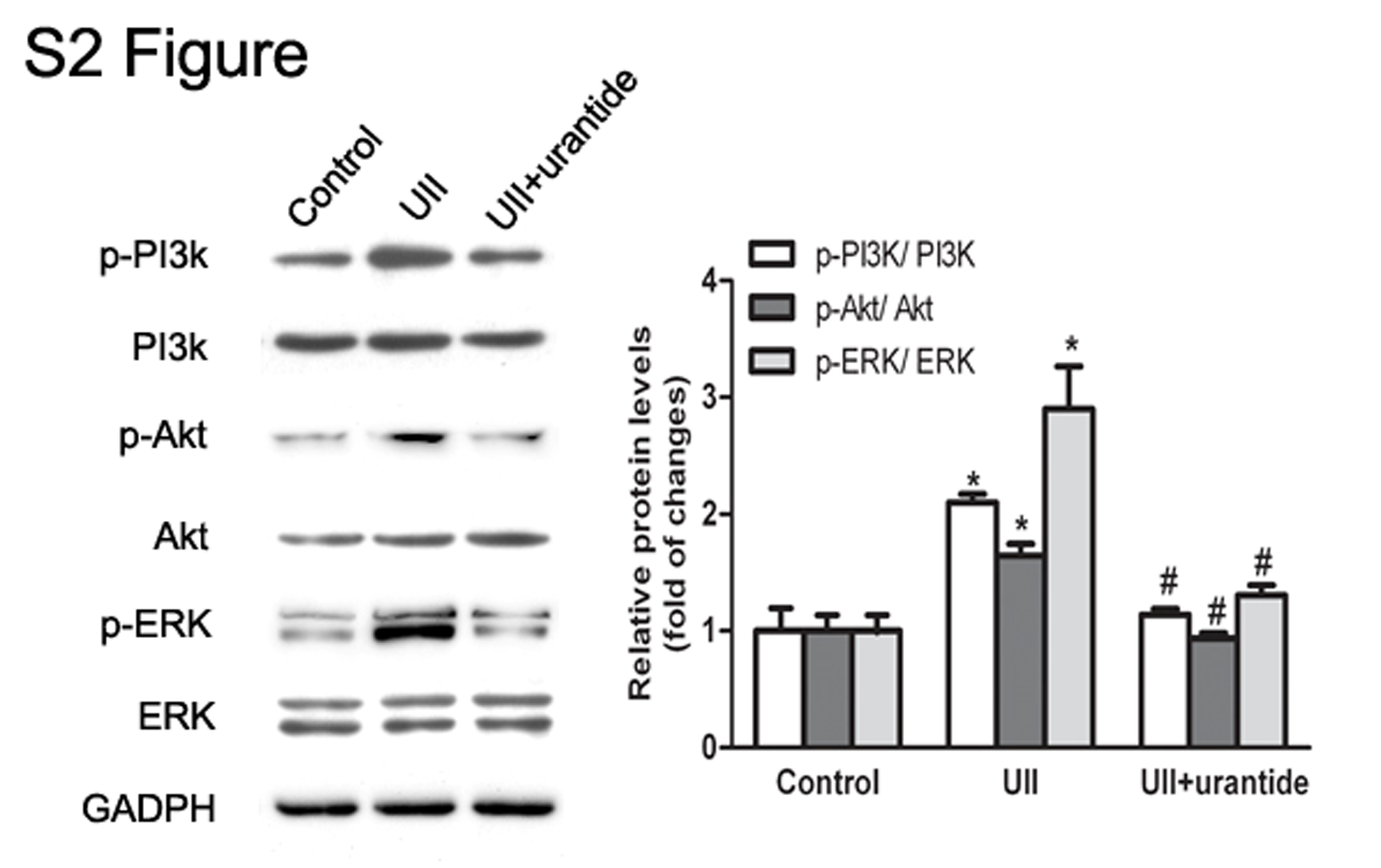

Supplement: S2 Fig — The protein levels were measured by western blotting and normalized against GADPH. After starvation, cells were treated with UII (10−9 M) for 2 h, with or without urantide pre-treatment for 30 min. Data are presented as mean ± SEM (n = 6). * P<0.05 versus control. # P<0.05 versus UII group. (TIF) [file pone.0144433.s002.tif]
